# First Clinical Description of Coagulation of Whole Blood with Resonant Acoustic Rheometry

**DOI:** 10.3390/diagnostics16010047

**Published:** 2025-12-23

**Authors:** Connor M. Bunch, Weiping Li, Kiera Downey, Timothy L. Hall, Allen Chehimi, Samuel J. Thomas, Afsheen Mansoori, Miguel Velasco, Marie N. Karam, Jenny Chen, Jacob Tuttle, Matthew R. Walsh, Scott G. Thomas, Mark M. Walsh, Joseph B. Miller, Jan P. Stegemann, Cheri X. Deng

**Affiliations:** 1 Departments of Emergency and Internal Medicine, Division of Critical Care Medicine, Henry Ford Health and Michigan State University Health Sciences, Detroit, MI 48202, USA; cbunch1@hfhs.org (C.M.B.); achehim2@hfhs.org (A.C.); jtuttle2@hfhs.org (J.T.); jmiller6@hfhs.org (J.B.M.); 2 Department of Biomedical Engineering, Michigan State University, East Lansing, MI 48824, USA; liweipi1@msu.edu; 3 Department of Biomedical Engineering, University of Michigan, Ann Arbor, MI 48109, USA; downeykl@umich.edu (K.D.); hallt@umich.edu (T.L.H.); jpsteg@umich.edu (J.P.S.); 4 Departments of Emergency and Internal Medicine, Saint Joseph Regional Medical Center, Mishawaka, IN 46545, USA; samueljthomas1@gmail.com (S.J.T.); markwalshmd@gmail.com (M.M.W.); 5 Departments of Emergency and Internal Medicine, Indiana University School of Medicine, South Bend, IN 46617, USA; afmans@iu.edu (A.M.); migvela@iu.edu (M.V.); markaram@iu.edu (M.N.K.); jc111@iu.edu (J.C.); 6 Equation 1 LLC, Mishawaka, IN 46544, USA; walsh.in@proton.me; 7 Department of Trauma & Surgical Research Services, Beacon Medical Group, South Bend, IN 46601, USA; sthomas@beaconhealthsystem.org

**Keywords:** acoustics, blood coagulation, critical illness, fibrinolysis, hemostasis, point-of-care systems, thromboelastography, ultrasonography, viscoelasticity

## Abstract

**Background/Objectives**: The timely evaluation of blood clot formation and breakdown is essential in the care of patients with severe bleeding or critical illness. Resonant acoustic rheometry is a novel, non-contact ultrasound method that measures changes in the viscoelastic properties of blood in a standard microplate format. Here, we present the first clinical description of whole blood coagulation and fibrinolysis assessed with resonant acoustic rheometry, with paired thromboelastography measurements for comparison. **Methods**: In this retrospective analysis, whole blood samples from three critically ill patients were divided and tested under four different conditions that included a control mixture, kaolin activation, tissue factor activation, and a tissue factor mixture supplemented with tissue plasminogen activator. The resonant acoustic rheometry system obtained real time measurements of resonant surface waves and displacements from the samples. Heat maps and spectrograms of the resonant surface waves were analyzed to determine the onset of clotting, the rate of viscoelastic stiffening, the time to maximum rigidity, and the onset as well as magnitude of fibrinolysis. These measurements were compared with thromboelastography reaction time, clot strength, fibrinogen contribution, and lysis values. **Results**: Resonant acoustic rheometry detected reproducible transitions from liquid to clot and from clot to lysis in all samples. Activator-dependent changes in clot initiation and propagation matched the expected hierarchy observed in thromboelastography. Significantly, samples exposed to tissue plasminogen activator demonstrated a clear fall in resonant frequency and a corresponding rise in surface displacement that reflected fibrinolysis. The technique also reproduced clinically meaningful patterns of hemostasis that aligned with each patient’s underlying disease. **Conclusions**: Whole blood clotting can be measured with resonant acoustic rheometry in a manner that aligns with established clinical assays. These results suggest strong potential for future use of resonant acoustic rheometry as a cost-effective, complementary platform for rapid, scalable, and clinically informative hemostatic assessment.

## 1. Introduction

The ability to quickly determine a patient’s relative tendency to clot or exsanguinate is important in emergency medicine, critical care, surgery, and combat injury. Point-of-care viscoelastic hemostatic assays (VHAs)—such as thromboelastography (TEG) and rotational thromboelastometry (ROTEM)—guide transfusion protocol and anticoagulant/antifibrinolytic dosage in these settings by assessing clot formation and fibrinolysis [1,2]. While VHAs have demonstrated use in trauma and surgical resuscitation, their sensitivity to fibrinolysis and certain coagulation abnormalities remains limited [3,4], and their high cost (of reagents/equipment) and low throughput (inability to perform large batch analyses) are further limitations [5]. Despite the wide adoption of VHAs and their clinical versatility, these shortcomings have fueled the search for a point-of-care test with low cost/high throughput potential, large batch analysis capacity, and increased information content.

We propose an ultrasound-based assay called resonant acoustic rheometry (RAR) as a candidate to efficiently and inexpensively quantify hemostatic competence during the entire lifespan of the clot from initiation, amplification, propagation, and termination through fibrinolysis. RAR is an emerging technology designed to assess blood coagulation by measuring viscoelastic properties in real time without direct sample contact or large deformation [6]. Employing standard labware such as a 96-well microplate, RAR provides rapid quantification of multiple samples simultaneously. The advantage of non-contact and minimally deforming techniques has been apparent in various manifestations [6,7,8,9,10,11,12,13,14,15], and indeed the TEG 6s is a light-emitting diode (LED)-based non-contact instrument which implements empirical correlations to recast temporal data as if from a cup-and-pin TEG 5000 [2]. The disadvantages of the TEG 6s include the requirement of specialized cartridge and high cost of the test chamber. RAR has been demonstrated in the characterization of plasma samples and model systems such as hydrogels [5,6,16]. These previous works described RAR’s success with plasma (non-lysing) and are summarized here:

- RAR showed the ability to identify a deficiency of clotting factor VIII in a plasma sample from a hemophilia A patient when compared to a control [16].
- RAR was able to identify fibrinogen concentration trends and predict hemostatic integrity in patients in diverse clinical situations such as liver, heart and kidney transplantation, and severe sepsis, and in bleeding trauma patients. In these patients, the RAR fibrinogen results were comparable to those obtained with the Clauss fibrinogen and TEG 6s functional fibrinogen [5].

RAR has not previously been deployed to identify the temporal and viscoelastic properties (hemostatic integrity) of a whole blood (WB) clot including initiation, amplification, propagation, and termination/fibrinolysis. This methods-advancement report and case series presents an introduction of RAR to the clinicians and its comparison to the VHA TEG, then presents the results from three critically ill patients experiencing severe hemorrhage who underwent simultaneous RAR and TEG analyses of WB. This series of patients represents the first successful description of the coagulation profile in a WB sample using RAR. We demonstrate successful determination of the four phases of the life span of a clot with the use of an adaptation of the plasma RAR to WB. Our results reveal a small-sample-size agreement between the RAR and the TEG when comparing the initiation of clot formation, and that in all three cases the expected trends based on additives to RAR WB experiments were followed: the addition of kaolin as in the Citrated Kaolin (CK) assay slightly decreased the time to clot vis-à-vis control, the addition of Tissue Factor as in the Citrated Rapid TEG (CRT) assay caused a more pronounced decrease in time to clot, and the addition of tissue plasminogen activator resulted in fibrinolysis (incidentally, the first achievement of lysis with RAR, necessitating a new variable to describe it, briefly previewed here in anticipation of data sufficient to allow the choice of a variable to describe termination/fibrinolysis).

The broader clinical implication of adopting RAR is its potential as a point-of-care approach to provide rapid, sensitive, and detailed hemostatic profiling at the bedside, enabling precise, personalized blood component therapies. Implementing RAR in clinical practice may facilitate timely, individualized transfusion strategies, ultimately improving patient outcomes, optimizing blood product use, and potentially reducing healthcare resource utilization and costs associated with management of severely bleeding patients [17].

## 2. Materials and Methods

### 2.1. Study Design and Participants

This retrospective study was approved by the Henry Ford Hospital Institutional Review Board (IRB No. 15804, date 30 August 2022). Three patients presenting with severe hemorrhage were included based on the availability of both WB TEG 6s and RAR measurements obtained during routine clinical care. Written informed consent has been obtained from the patients to publish this paper.

### 2.2. Blood Sample Collection and Processing

WB samples were collected in blue-top Vacutainer^®^ tubes containing 0.109 M (3.2%) trisodium citrate following Clinical Laboratory Standards Institute (CLSI) guidelines. Samples were immediately processed for TEG and RAR analysis to minimize pre-analytical variability.

### 2.3. Thromboelastography (TEG 6s) Analysis

Citrated WB was incubated for 10 min, and the samples were assayed at room temperature using a TEG 6s Global Hemostasis^®^ cartridge or a Global Hemostasis with Lysis^®^ cartridge (Haemonetics Corporation, Braintree, MA, USA) at clinician’s discretion. The Global Hemostasis^®^ cartridge contains four assay channels with different dried activators/inhibitors. The four channels are the following: 1. Citrated Kaolin (CK) using kaolin 0.004% *w*/*w* and CaCl_2_ 0.9 M; 2. Citrated Rapid TEG (CRT) using kaolin 2% *w*/*w*, Tissue Factor 2 μg/mL, and CaCl_2_ 0.8 M; 3. Citrated Kaolin with Heparinase (CKH) using kaolin 0.007% *w*/*w*, CaCl_2_ 0.9 M, and heparinase ≥ 400 IU/mL; and 4. Citrated Functional Fibrinogen (CFF) using Tissue Factor 0.3 μg/mL, CaCl_2_ 0.8 M, and abciximab 2 mg/mL [15]. The Global Hemostasis with Lysis^®^ cartridge contains three assay channels: 1. CK, 2. CRT, and 3. CFF. Maximum amplitude (MA) (reference range 52–70 mm), CFF MA (also referred to as MA-FF) (reference range 16–32 mm), reaction time (R, reference range 4.6–9.1 min), and lysis at 30 min (LY30, reference range 0.0–2.6%) were the quantitative measurements obtained from the TEG 6s assays. An elevated LY30 indicates hyperfibrinolysis, which has a high clinical incidence in the anhepatic phase of liver transplantation and is also seen in severe injury with trauma-induced coagulopathy [5,18,19,20,21,22].

### 2.4. RAR System and Operation

A prototype RAR system as described previously was used in this study [5]. Briefly, the RAR system employed a composite ultrasound transducer assembly that includes two concentric and co-linearly aligned focused ultrasound transducers. The outer, annular transducer (center frequency 1.5 MHz) was used as the excitation transducer, and the inner transducer (center frequency 7 MHz) worked as the detection transducer. The excitation transducer, driven by a power amplifier (75A250; Amplifier Research Inc., now AR RF/Microwave Instrumentation, AMETEK, Souderton, PA, USA) and a waveform generator (33220 A; Agilent Technologies, now Keysight Technologies, Santa Rosa, CA, USA), was used to generate a short ultrasound pulse to induce perturbation on the top surface of the sample. The detection transducer, controlled by a pulser/receiver (5900PR; Olympus, Waltham, MA, USA), was used to operate in a pulse-echo mode to probe the movement of the sample surface based on the temporal shifts of the echo signals from the sample surface before, during, and after the initial perturbation. The received echo signals were digitized using Picoscope (model 5443, Pico Technology, St Neots, UK). The excitation and detection transducers were synchronized with a pulse generator (Model 565; Berkely Nucleonics Corporation, San Rafael, CA, USA) for both excitation and tracking of motion on the sample surface [5].

For RAR testing, each sample was placed in one well of a 96-well microplate, which was halfway submerged in a room temperature water bath for acoustic coupling. The ultrasound transducer assembly was placed below, completely submerged, facing upward with the foci placed on the center of the top surface of the sample. Multiple samples were housed in different wells of the microplate. Multiplexing operation was implemented such that the RAR transducer was aligned and positioned, using a three-dimensional motion control platform (UniSlide, Velmex Inc., Bloomfield, NY, USA) to enable measurement of multiple samples housed in the microplate.

### 2.5. RAR Assays of WB Samples

All citrated WB samples were divided into four equal portions for four RAR reagent conditions, which contained different concentrations of CaCl_2_ (Fluka, Honeywell, Charlotte, NC, USA) Rabbit Brain Cephalin (Lipids, Pel-Freez Biologicals, Rogers, AR, USA), kaolin (Fisher Scientific, Waltham, MA, USA), Tissue Factor stock (10× Dade Innovin, Siemens Healthineers, Erlangen, Germany), recombinant tissue plasminogen activator from humans (tPA, Sigma-Aldrich, St. Louis, MO, USA), and 0.85% buffered saline (Fisher Scientific). The four reagent conditions are the following: 1. Control (calcium 15 mM, lipids 25 μM); 2. CK (calcium 15 mM, lipids 25 μM, and kaolin 0.004% *w*/*w*); 3. CRT (calcium 15 mM, lipids 25 μM, kaolin 2% *w*/*w*, and TF 2 μg/mL); 4. CRT + tPA (calcium 15 mM, lipids 25 μM, kaolin 2% *w*/*w*, TF 2 μg/mL, and tPA 2 μg/mL).

For each RAR assay, four 90 μL WB samples were mixed with 10 μL of their respective reagent mix, and each of the 100 μL mixtures were then quickly pipetted into one well of the 96-well plate. RAR commenced 20 s after the WB and reagents were mixed [5].

### 2.6. Measurements of Resonant Surface Waves in WB Samples with RAR

After the initial surface perturbation by the excitation ultrasound pulse, a resonant surface wave was formed on the top sample surface, which is detected by the detection transducer operated at pulse-echo mode at high pulse repetition frequency of 5 KHz to capture the dynamic surface movement. At each time point after the excitation, measurements of the surface displacement in the sample was determined from the temporal shifts of the echo signals received by the detection transducer using the equation $d=\frac{c∆t}{2}$, where c is the speed of sound in the medium (water) and ∆t is the shift in the arrival time of the echo signals from those before surface perturbation. The MATLAB R13 Phase Unwrap command was used across sequential phase measurements to allow for accurate displacement measurements beyond a single acoustic cycle (approximately 214 μm), if the max velocity was less than one half-cycle per imaging pulse period (approximately 0.5 m/s). The entire surface movement after each excitation was then determined from the temporal shifts of all the echo signals after excitation at high temporal resolution of 0.0002 s.

To determine the power spectrum of the surface movement, a Fast Fourier transform of the measured dynamic surface displacement data was performed [5,6,16]. For RAR measurement of a coagulating sample, repeated excitation and detection of the surface movement or resonant surface waves were performed at a time interval of 3–5 s to detect the surface movements and assess the property of the sample during coagulation.

The ensemble of the surface displacements measured over time during coagulation was presented in an image format as surface displacement heat map; the amplitude was color-coded with the vertical axis representing the dynamic surface movement after each excitation and the horizontal axis representing the coagulation time. The collection of spectra of the resonant surface waves was also presented in an image format as spectrogram with the vertical axis representing the frequency of the surface wave after each excitation and the horizontal axis representing the coagulation time. The spectrograms measured using RAR were used to extract quantitative parameters to compare with TEG.

### 2.7. Similarities Between TEG and RAR

**Figure 1:**
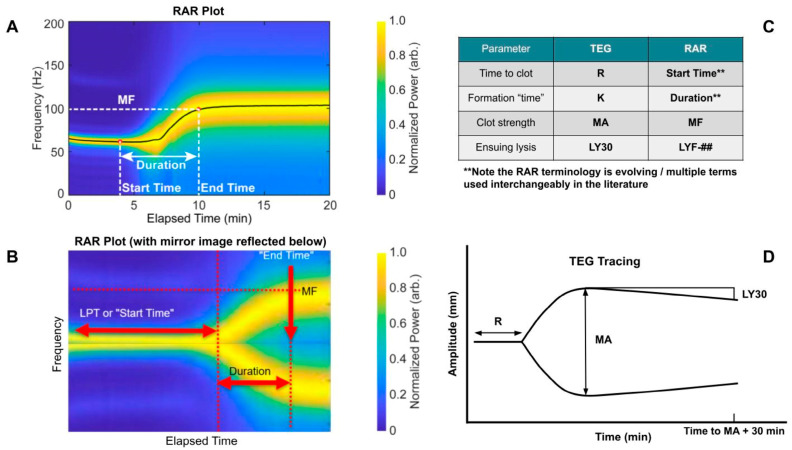
Introduction of thromboelastography (TEG) and resonant acoustic rheometry (RAR) measurements from previous plasma work. (**A**) Representative RAR spectrogram. (**B**) Mirror image of RAR spectrogram to show similarity with TEG tracing. (**C**) Analogous TEG and RAR measurements. (**D**) Representative TEG tracing. Note the analog of clot kinetics (K) and Duration is not exact due to the inability of obtaining reliable RAR data at such fine time intervals. Also note that in RAR, the Start Time is defined by a drop in the max displacement heat map, which is not shown here for simplicity but described further below. Note that lysis at 30 min (LY30) on the TEG is defined as the percent decrease in the amplitude 30 min following the maximum amplitude (MA). This measurement may also be obtained at different time intervals such as LY60. The TEG manufacturer, Haemonetics, has standardized the % lysis measurement on the newer generation TEG 6s device to LY30, so that is what is shown here for simplicity. For RAR, it was purposefully left undefined, as represented by the ##, at which time point lysis frequency (LYF) will be measured since it is currently determined by the operator, akin to the legacy cup-and-pin TEG 5000 device. For example, LYF-30 would represent the % decrease in frequency at 30 minutes after reaching maximum frequency (MF).

RAR excites and measures resonant surface waves in a sample housed in a standard labwave such as a 96-well microplate in a non-contact fashion. Since the frequency of the resonant surface waves is directly related to the viscoelastic properties of the sample through the dispersion relation, RAR measurements of the resonant surface wave frequency provide a rigorous approach for viscoelastic quantification. Conveniently, increased resonant frequency in RAR represents an increase in sample stiffness, while the width of the resonant frequency peak reflects the viscosity of the sample. A spectrogram in RAR readily captures the changing resonant frequency over time, representing the changing viscoelastic property of a coagulating sample as shown in Figure 1. In addition, the amplitude of the resonant surface wave also sensitively captures the stiffness of the sample, as stiffer samples produce surface waves with smaller amplitudes, as stiffer samples are expected to exhibit smaller deformation when the deforming ultrasound pulse is kept constant during a measurement session with RAR. Thus, a heat map of the RAR surface wave amplitude is also used to provide a visual representation of a clotting sample over time. Thus, both the RAR spectrogram and RAR displacement heat map obtain sensitive characterization of blood coagulation in real time, where the frequency rapidly increases and the displacement amplitude decreases, and of fibrinolysis, where the frequency decreases and the displacement amplitude increases.

As a reference for understanding the graphical representation of initiation, amplification, propagation, and termination through fibrinolysis of WB samples utilizing RAR, Figure 1 is noted below.

The original TEG (TEG 5000) operated with a cup-and-pin setup in which amplitude correlated with clot strength [2]. The TEG 6s implements LED (meaning frequency would be the parameter to correlate to clot strength as in RAR), yet through exhaustive empirical correlations translates the data to appear as if obtained from a cup-and-pin apparatus in which amplitude would correlate to clot strength [2]. As the RAR is new—precluding such a correlation—we present the frequency “shape” on the RAR plots compared to the amplitude “shape” on the TEG plots and note the similarities, as mirroring the RAR (Figure 1B) appears like a TEG plot (Figure 1D), a promising sign for RAR’s potential as a low-cost/high-throughput alternative to TEG.

The main objectives of this paper are to show that WB clotting was detectable using RAR for the first time. To provide context of this new study, we summarize below the RAR suite of measurements used in this study in relevance to those used in our previous plasma assay.

- Start Time (the time at which a clot starts to form, measured most precisely by a drop in max displacement on the heat maps)
- MF (Maximum Frequency)
- Duration (time required to reach MF after start time)
- LYF-## (Lysis Frequency; Some time measure of post-MF lysis akin to LY30)
- Note that Frequency Change (FC) may be useful as a measurement itself if the initial frequency shows appreciable variation from experiment to experiment or because, while a maximum is important, where it started from is also salient [16].
- Given the importance of WB assay, this report focuses on quantification of RAR measurements in terms of Start Time, which was related to the Liquid Phase Time used in our previous plasma characterization. For WB (which can lyse), the “Final Resonant Frequency” will no longer be appropriate.

**Figure 2:**
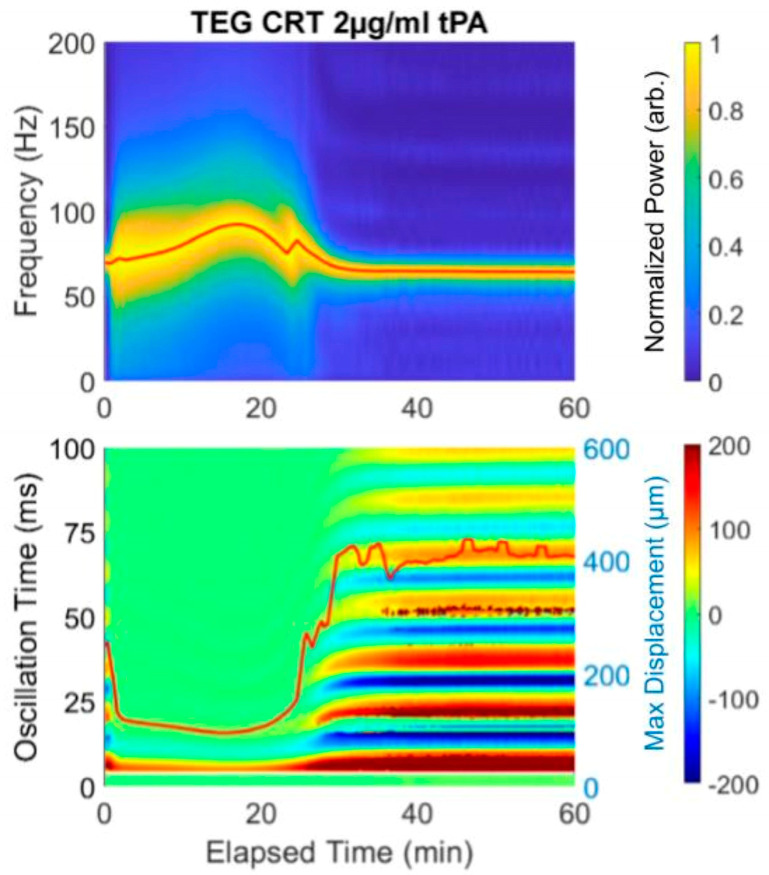
Example of an RAR spectrogram and a heat map of the surface displacement below for the same experiment showing near immediate clotting (decrease in max surface displacement and limiting of the multiple oscillation times, followed by lysis (drop in frequency and increase in max displacement). Notice that the initiation of clotting, indicated as the significant widening of the plot in the spectrogram at 4 min, corresponds exactly to the transition from the liquid phase to solid phase, which is represented by the rapid change in the surface displacement amplitude shown in the heat map from red/blue into (predominantly) green.

Importantly, we provide data-rich heat maps of surface wave amplitude (shown in Figure 2). Heat maps plot measured oscillation times vs. elapsed time with max surface displacement, showing the temporal evolution of the surface waves in the sample, which also provide complement information for determining start time from the spectrogram. The max displacement decreases when a clot is formed compared to that in liquid and increases again upon lysis.

The spectrogram and heat map in Figure 2 reveal lysis with the addition of tPA. Because this sample was analyzed with the addition of Tissue Factor, the Start Time is very short, as it is in the rapid TEG with Tissue Factor as an activator. Therefore, the clot forms nearly immediately and the Duration time until MF is 20 min, at which point the frequency is 100 Hz. Subsequent spiking with 2 µg/mL tPA causes a drop in the final resonant frequency from 100 to approximately 75. In this prototype, a concentration of 2 µg/mL tPA was used to ensure lysis. The equivalent of the LY30 would be the F at 50 min, which is 75 Hz. The heat map reflects the lysis which begins at 20 min.

## 3. Results

### 3.1. Case Histories

**Figure 3:**
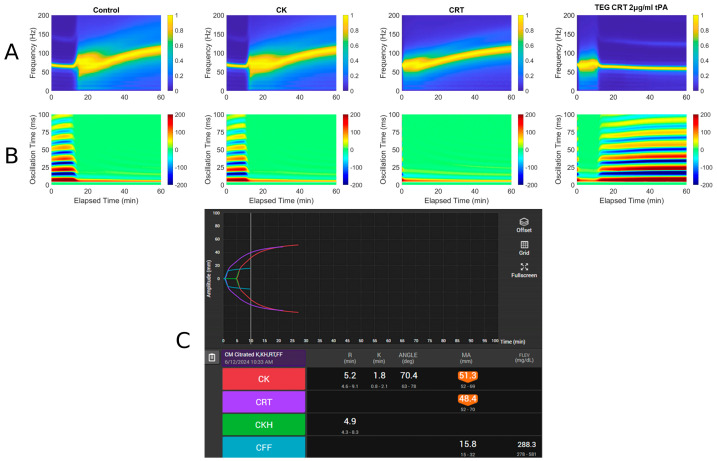
RAR spectrogram and displacement heat map vs. TEG in Case # 1. (**A**) Spectrograph of control, kaolin TEG (CK), rapid TEG (CRT), and rapid TEG tPA challenge test (TEG CRT 2 µg/mL tPA). (**B**) Heat map of control, kaolin TEG (CK), rapid TEG (CRT), and rapid TEG tPA challenge test (TEG CRT 2 µg/mL tPA). (**C**) TEG 6s of patient 1. Kaolin TEG (CK), rapid TEG (CRT), kaolin heparinase TEG (CKH), functional fibrinogen (CFF), reaction time (R), clot kinetics (K), alpha angle (ANGLE), maximum amplitude (MA), functional fibrinogen (FLEV).

**Figure 4:**
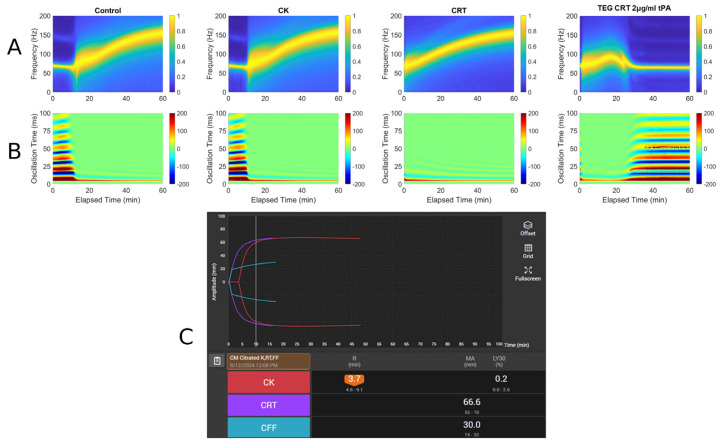
RAR spectrogram and displacement heat map vs. TEG in Case # 2. (**A**) Spectrograph of control, kaolin TEG (CK), rapid TEG (CRT), and rapid TEG tPA challenge test (TEG CRT 2 µg/mL tPA). (**B**) Heat map of control, kaolin TEG (CK), rapid TEG (CRT), and rapid TEG tPA challenge test (TEG CRT 2 µg/mL tPA). (**C**) TEG 6s of patient 2. Kaolin TEG (CK), rapid TEG (CRT), kaolin heparinase TEG (CKH), functional fibrinogen (CFF), reaction time (R), clot kinetics (K), alpha angle (ANGLE), maximum amplitude (MA), functional fibrinogen (FLEV).

**Figure 5:**
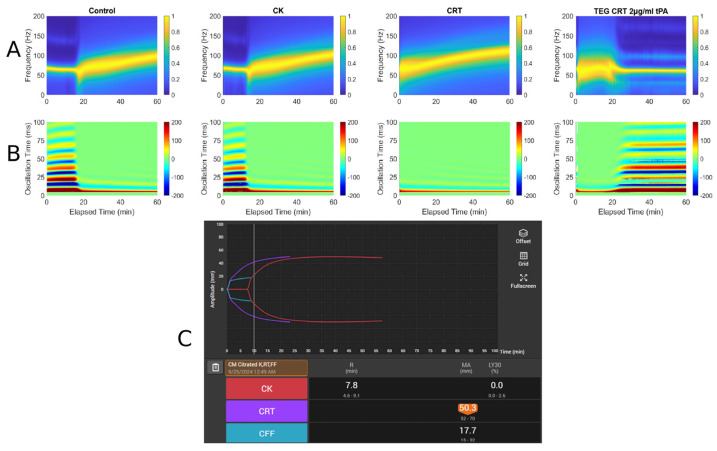
RAR spectrogram and displacement heat map vs. TEG in Case # 3: (**A**) Spectrograph of control, kaolin TEG (CK), rapid TEG (CRT), and rapid TEG tPA challenge test (TEG CRT 2 µg/mL tPA). (**B**) Heat map of control, kaolin TEG (CK), rapid TEG (CRT), and rapid TEG tPA challenge test (TEG CRT 2 µg/mL tPA). (**C**) TEG 6s of patient 3. Kaolin TEG (CK), rapid TEG (CRT), kaolin heparinase TEG (CKH), functional fibrinogen (CFF), reaction time (R), clot kinetics (K), alpha angle (ANGLE), maximum amplitude (MA), functional fibrinogen (FLEV).

Three case histories with associated RAR spectrographs and heat maps with accompanying TEG 6s tracings were used. The abbreviations for Figure 3, Figure 4 and Figure 5 are listed below.

- A: Spectrograph of control, kaolin TEG (CK), rapid TEG (CRT), and rapid TEG tPA challenge test (TEG CRT + 2 µg/mL tPA)
- B: Heat map of control, kaolin TEG (CK), rapid TEG (CRT), and rapid TEG tPA challenge test (TEG CRT + 2 µg/mL tPA)
- C: TEG 6s of patient 1. Kaolin TEG (CK), rapid TEG (CRT), kaolin heparinase TEG (CKH), functional fibrinogen (CFF), reaction time (R), clot kinetics (K), alpha angle (α), maximum amplitude (MA)

#### 3.1.1. Case # 1

A 61-year-old male with a history of decompensated alcoholic cirrhosis (Model for End-Stage Liver Disease score = 28, Child–Pugh C), coronary artery disease (CAD), chronic thrombocytopenia, and chronic obstructive pulmonary disease requiring home oxygen, was undergoing evaluation for liver transplantation when he was diagnosed with significant CAD. Due to his thrombocytopenia and history of esophageal varices, he was considered a poor candidate for percutaneous coronary intervention (PCI), as he could not tolerate dual antiplatelet therapy (DAPT). A prior DAPT trial was discontinued after he developed epistaxis and rectal bleeding, prompting his surgical team to recommend high-risk coronary artery bypass graft (CABG). On day of admission, the patient underwent off-pump CABG with a right internal mammary artery to right coronary artery bypass. He experienced excessive intraoperative bleeding, requiring transfusion of 4 units of fresh frozen plasma (FFP) and 2 pools of platelets (PLT). He was then transferred to the cardiovascular intensive care unit while intubated and sedated and then was successfully extubated within six hours. Postoperatively, he developed multiple complications, including an acute kidney injury, hyponatremia, atrial fibrillation with rapid ventricular response, a large hematoma at the median sternotomy site, recurrent cardiac tamponade for which he had to emergently go back to the operating room for mediastinal washout, and intubated with four-vasopressor dependent shock. On the day of TEG (Figure 3), blood product administration was adjusted in which the patient received 1 unit of packed red blood cells, 1 FFP, and 1 pool PLTs as he exhibited persistent drainage from the median sternotomy site. Labs were remarkable for a platelet count of 65 × 10^9^/L, fibrinogen of 176 mg/dL, and INR of 2.97. Despite ongoing resuscitative efforts, his condition continued to deteriorate. Fourteen days after surgery, a CT scan revealed a moderate-to-large volume of intra-abdominal blood without evidence of active extravasation, and bleeding from his right and mediastinal chest tubes with volume outputs exceeding 500 mL an hour. Surgical intervention was deemed futile, the family ultimately withdrew care, and the patient expired shortly thereafter.

#### 3.1.2. Case # 2

The next case is a 68-year-old male with a complicated history of CAD status post-PCI, ischemic cardiomyopathy with severely reduced left ventricular ejection fraction of 32% and an automated implantable cardioverter–defibrillator, venous thromboembolism status post-inferior vena cava filter placement, type 2 diabetes mellitus, esophageal stricture requiring serial dilations complicated by perforation, *Candida glabrata* and *Enterobacter cloacae* empyema, gastrointestinal bleed requiring coil embolization of the jejunal branch of the superior mesenteric artery, and ventilator-dependence due to chronic respiratory failure with failure to wean status post tracheostomy and percutaneous gastrostomy. The patient was initially evaluated at an outside hospital, with complaints of abdominal distention, nausea, vomiting, and abdominal pain. Imaging revealed bilateral hydropneumothorax, multifocal pneumonia, and subsegmental pulmonary emboli. He was started on broad-spectrum antibiotics (cefepime, metronidazole, micafungin) and a heparin drip. Upon admission to the medical intensive care unit, he exhibited signs of worsening respiratory distress and persistent anemia. Bilateral thoracostomies were placed for bilateral pleural fluid collections and empyema, with pleural fluid cultures growing vancomycin-resistant Enterococcus, treated with intravenous Linezolid for four weeks. TEG and RAR were obtained on his day of admission when his hemoglobin was 5.3 g/dL without any overt signs of hemorrhage (Figure 4). Coagulation labs included a platelet count at 189 × 10^9^/L, INR of 1.46, PTT of 29, PT of 17.4; fibrinogen was not measured. Throughout his hospitalization, the patient required intermittent transfusions of packed red blood cells, although the patient did not exhibit overt hemorrhage or active gastrointestinal bleeding. Given his negative presentation for active gastrointestinal bleeding, an esophagogastroduodenoscopy was performed and reassured findings of no active upper gastrointestinal bleeding. His hospital course was further complicated by persistent empyema requiring prolonged thoracic drainage, multiple subsegmental pulmonary emboli, and recurrent hospital-acquired pneumonia. He remained critically ill with progressive respiratory failure and multi-organ dysfunction. He was ultimately transitioned to comfort measures and discharged to a long-term acute care hospital for continued supportive care. Figure 4 demonstrates a common manifestation of hypercoagulability of severe sepsis, as demonstrated by heparin resistance, the presence of pulmonary emboli, and a short R interval in the TEG.

#### 3.1.3. Case # 3

A 58-year-old male with a history of alcohol use disorder, decompensated cirrhosis, hypertension, diabetes mellitus, thrombocytopenia, esophageal varices, colonic arteriovenous malformations, and portal gastropathy presented to the emergency department after a mechanical fall followed by a generalized tonic–clonic seizure. The patient reported that his legs gave out, causing him to strike the left side of his head on the ground. On arrival at the emergency department, he was neurologically intact with a Glasgow Coma Scale of 15. Physical examination revealed left periorbital ecchymosis, a left posterior scalp hematoma, and tenderness over the left humerus. A non-contrast CT of the head demonstrated acute on chronic right-sided subdural hematoma measuring 6 mm in thickness with a leftward midline shift of 4 mm, a subacute left-sided subdural hemorrhage measuring 5 mm, scattered subarachnoid hemorrhages along the right temporal lobe and Sylvian fissure, and an intraparenchymal hemorrhage in the right basal ganglia measuring 13 × 8 × 4 mm. A moderate-sized left parietal scalp hematoma was also noted, though there were no acute skull fractures. He was admitted to the surgical intensive care unit for close neurological monitoring. A repeat CT scan at 24 h showed an increase in the size of the right basal ganglia hemorrhage to 18 × 12 mm, evolving right temporal hemorrhagic contusions, and progression of the subdural hematomas bilaterally. Midline shift remained stable at 4 mm. Lab workup demonstrated thrombocytopenia with a PLT count of 82 × 10^9^/L, a fibrinogen level of 305 mg/dL, INR of 1.27, PT of 15.9, and PTT of 29. TEG and RAR performed on hospital day 2 demonstrated low MA suggesting PLT dysfunction being the primary factor contributing to the patient’s increased bleeding risk. Neurosurgery recommended TEG-directed transfusions, with a PLT transfusion goal of >100 × 10^9^/L. Patient ultimately remained clinically stable and was discharged without new neurological deficits. Figure 5 demonstrates a very narrow MA, which indicates a decrease in PLT functionality, a well-known complication of TBI [23].

### 3.2. TEG and RAR for WB

Taken together, these results show clotting of WB in controls and in severely coagulopathic patients using RAR methodology which has previously been described only in plasma samples and model hydrogels. Using standard reagents for the TEG 5000 auto-analyzer, we demonstrated RAR for capturing both clot formation and lysis. In addition, we detected the presence of fibrinolysis precipitated by spiking the samples with recombinant tPA. Spectroscopy and accompanying heat map data of RAR measurements of resonant surface waves in the samples demonstrate a progressively increasing hypercoagulable state from the activator-deficient control to the kaolin CK and to the kaolin + Tissue Factor rapid assay CRT.

These observations of tPA challenge test using RAR are consistent with those of WB in thromboelastographic using TEG, as well as ROTEM experimental models [24,25].

**Figure 6:**
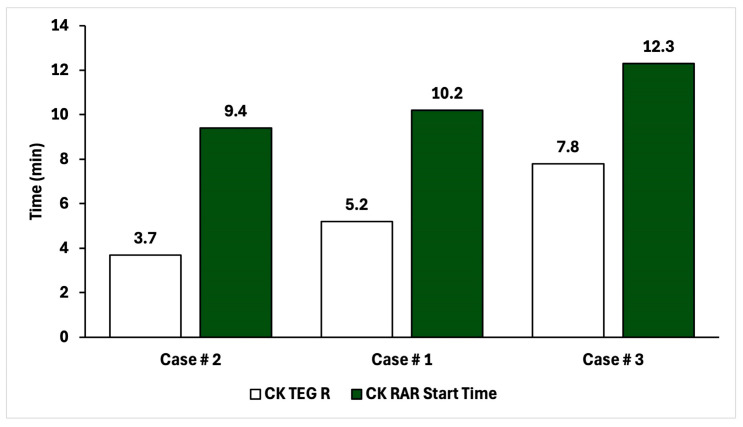
Comparison of time-to-clot for CK TEG and CK RAR for the three patients in this study, in ascending order.

It is also promising that the rankings of time-to-clot aligned between RAR and TEG for the assay for which complete data was possible (CK). Figure 6 shows RAR Start Time vs. TEG R for the CK assay condition. In all three cases, the RAR Start Time captures the same trend as TEG R, although significantly higher values are obtained in RAR than TEG. This could be due to systematic differences in measurement protocols between RAR and TEG where mixing of reagents with WB might be an issue to investigate further. The performance in these three experiments of RAR as a replacement for TEG is quantitatively striking.

## 4. Discussion

Previously, the RAR technique successfully demonstrated genetic and acquired coagulation deficiencies in diverse clinical situations of bleeding of coagulation factors and fibrinogen [5,16]. The findings in this proof-of-concept case series confirm the validity of the novel RAR WB methodology as a reliable approach system for assessing coagulation and fibrinolysis. The three patients represent the first successful demonstration of the RAR to detect coagulopathy disorders in WB preparations. Our results demonstrate a consistent trend with previous plasma-based studies, with the resonance frequency shifts corresponding to the four classical stages of hemostasis: initiation, amplification, propagation, and lysis. The consistency of clot formation across all test conditions, coupled with the expected acceleration in clotting with CK and CRT assays, supports the robustness of this technique in WB characterizations.

All cases demonstrated a progression of clot formation with the addition of activator kaolin and tissue factor as exemplified by the fact that the sample with CK clotted faster than the control, and that the sample with CRT clotted faster than that with CK. In addition, the tPA-spiked CRT sample demonstrated immediate clotting followed by lysis when tPA was added. These clotting trends demonstrate the ability of the RAR WB assay to accurately reflect hemostatic competence during the initiation, amplification, propagation, and termination of coagulation in patients with severe bleeding and clotting disorders.

These three cases of patients with coagulopathy caused by decompensated cirrhosis, of ischemic cardiomyopathy associated with gastrointestinal bleeding, and a patient with coagulopathy of traumatic brain injury, demonstrate the ability of this methodology to accurately reflect the hemostatic competence of the patient as represented by the TEG 6s.

These results are significant because they confirm the validity of previous plasma samples tested by RAR which demonstrated similar correlation between pulse ultrasound and time, as well as resonant frequency and time as a reflection of the four stages of clot initiation, amplification, propagation, and lysis. The similarities between plasma and WB samples confirm the validity of the methodology. In WB, there was a dramatic and noticeable drop in the final resonance frequency on the spectroscopy and heat map which reflects the importance of the participation of not just coagulation in fibrin in clot formation, but also fibrin crosslinking and platelet–fibrin contraction during amplification and propagation. This phenomenon further confirms the paradigm shifting concept of the cell-based theory of hemostasis as promulgated by Hoffman and Monroe et al. 2001, emphasizing the critical interplay between coagulation factors, fibrin crosslinking, and platelet-mediated contraction [26].

One of the limitations of this article is the small sample size of three patients. Future studies with more patients of differing pathologies are planned to apply the observations of this first-time use of RAR with WB samples for determining hemostatic competence.

In practical terms, the RAR WB assay represents a significant advancement due to its adaptability, high throughput, and lower cost compared to traditional methodologies. Moreover, RAR offers customizability for single or multiple specimens for hemostatic competence in emergency conditions. The ability to use commercially available reagents compatible with the TEG 5000 further enhances its utility for widespread adoption.

## 5. Conclusions

In this paper, we show for the first time that WB clotting can be assessed with RAR, RAR trends are consistent within additive-based-expectations, and the times-to-clot correlate between RAR and TEG.

The cases presented demonstrate the applicability of RAR in diverse coagulopathic states, including liver disease, trauma, and cardiovascular pathology. Future studies are planned to refine the methodology by incorporating larger datasets and demonstrate investigating potential clinical correlations with clinical test results and outcomes to further establish the clinical relevance of RAR WB testing in guiding hemostatic management and therapeutic interventions.

## Data Availability

The data presented in this study are available on request from the corresponding author. The data are not publicly available due to privacy, legal, and ethical restrictions.

## Abbreviations

The following abbreviations are used in this manuscript:

CABG	coronary artery bypass graft
CAD	coronary artery disease
CFF	Citrated Functional Fibrinogen
CFS	clot formation speed
CK	Citrated Kaolin
CKH	Citrated Kaolin with Heparinase
CLSI	Clinical Laboratory Standards Institute
CRT	Citrated Rapid Thromboelastography
DAPT	dual antiplatelet therapy
FC	frequency change
FCR	frequency change rate
FFP	fresh frozen plasma
K	clot kinetics
LY30	lysis at 30 min
LYF	lysis frequency
MA	maximum amplitude
MF	maximum frequency
PCI	percutaneous coronary intervention
PLT	platelets
R	reaction time
RAR	resonant acoustic rheometry
ROTEM	rotational thromboelastometry
TEG	thromboelastography
tPA	tissue plasminogen activator
VHA	viscoelastic hemostatic assay
WB	whole blood
